# Regulation of immunogenic cell death and potential applications in cancer therapy

**DOI:** 10.3389/fimmu.2025.1571212

**Published:** 2025-03-26

**Authors:** Kun Fang, Shuai Yuan, Xue Zhang, Jingdong Zhang, Shu-lan Sun, Xiaoxi Li

**Affiliations:** ^1^ Central Laboratory, Cancer Hospital of Dalian University of Technology, Cancer Hospital of China Medical University (Liaoning Cancer Hospital & Institute), Shenyang, Liaoning, China; ^2^ Liaoning Key Laboratory of Gastrointestinal Cancer Translational Research, Shenyang, Liaoning, China; ^3^ Department of Medical Oncology, Cancer Hospital of Dalian University of Technology, Cancer Hospital of China Medical University (Liaoning Cancer Hospital & Institute), Shenyang, Liaoning, China

**Keywords:** immunogenic cell death, DAMPs, immunotherapy, ER stress, cancer, synergic therapy

## Abstract

Immunogenic cell death (ICD), a type of regulatory cell death, plays an important role in activating the adaptive immune response. Activation of the tumor-specific immune response is accompanied by the cell surface exposure of calreticulin and heat-shock proteins, the secretion of adenosine triphosphate, and the release of high mobility group box-1. In this review, we summarize and classify the latest types of ICD inducers and their molecular mechanisms, and discuss the effects and potential applications of inducing ICD by chemotherapy drugs, targeted drugs, and oncolytic viruses in clinical research. We also explore the potential role of epigenetic modifiers in the induction of ICD, and clarify the synergistic anti-tumor effects of nano-pulse stimulation, radiosensitizers for radiotherapy, photosensitizers for photodynamic therapy, photothermal therapy, and other physical stimulation, combined with radiotherapy and chemotherapy induced-ICD, in multimodal immunotherapy. In addition, we elucidate the molecular mechanism of ICD in detail, including the calcium imbalance, mitochondrial stress, and the interactions in the tumor microenvironment. Ultimately, this review aims to offer deeper insight into the factors and mechanisms of ICD induction and provide a theoretical basis for the future development of ICD-based immunotherapy.

## 1 Introduction

Immune surveillance plays an important role in the occurrence, development, and prognosis of tumors. As the body’s first line of defense against cancer, immune surveillance can effectively identify and eliminate emerging cancer cells. However, with further mutation and adaptation, some cancer cells can evade immune surveillance, resulting in tumor invasion, metastasis, and a poor prognosis (1, 2). To restore or enhance the immune surveillance ability of the body, in recent years, immunotherapeutic drugs represented by immune checkpoint inhibitors (e.g., anti-PD-1/anti-PD-L1) have been widely used in clinic, which has completely changed the treatment prospects for many diseases (3). Although immunotherapy with PD-1 and PD-L1 has benefited many patients, data show that only 10%–58% of patients (depending on the tumor species) respond positively to such immunotherapy (4). Studies indicate that immune cells near to tumor tissue exhibit strong immunogenicity and infiltrate the tumor, transforming it into a “hot tumor”, and inducing tumor cells to undergo immunogenic cell death (ICD), which seems to be an effective means to enhance tumor therapy (5, 6). It is this immune-transformation of the tumor that is targeted in the curative effect of PD-1. In clinical trials, compared with immunotherapy alone, chemotherapy combined with immunotherapy can significantly improve the objective response rate and the prognosis of tumor patients (7–10). Therefore, an in-depth understanding of the factors and regulatory mechanisms that may induce ICD is expected to transform non immunogenic “cold tumors” into immunogenic “hot tumors,” thereby enhancing the therapeutic efficacy of immune checkpoint inhibitors. This would enable more patients to benefit from immunotherapy, prolong their survival, and improve their quality of life.

**Figure 1:**
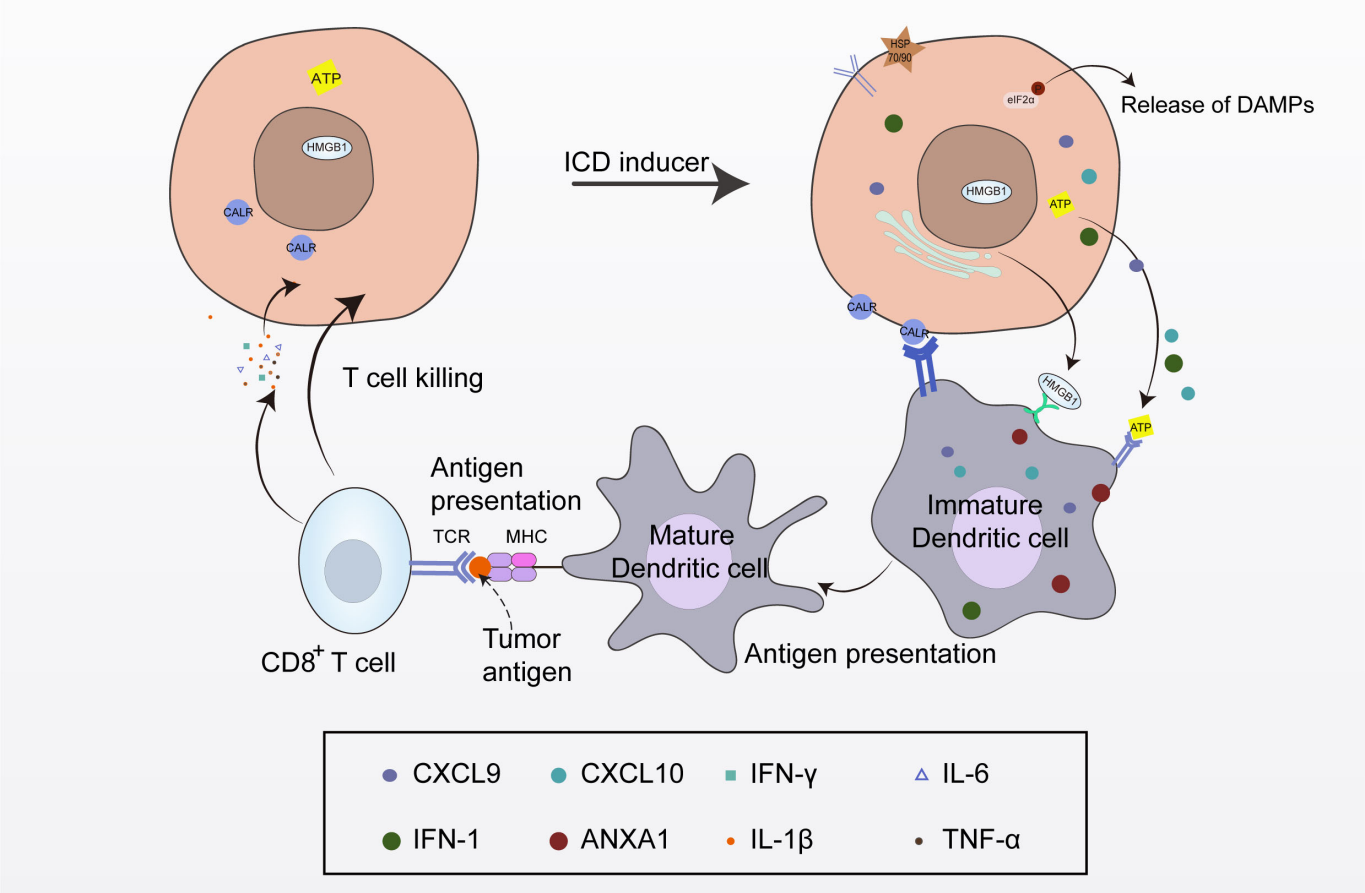
The process of immunogenic cell death (ICD). Following the induction of ICD, dying tumor cells release tumor antigens and damage-associated molecular patterns (DAMPs). DAMPs stimulate pattern recognition receptors on dendritic cells (DCs), leading to DC maturation, T cell activation, and the initiation of tumor-specific anti-tumor immune responses.

ICD refers to the death of tumor cells triggered by external stimuli, which induce the release of danger signals that transform non-immunogenic cells into immunogenic cells, thereby mediating an anti-tumor immune response (11, 12). This is a widely regulated form of cell death that can activate the body’s adaptive immune response (12). Cancer cells undergoing ICD regulate their microenvironment via the following factors: active exposure to specific proteins, such as calreticulin (CALR), protein disulfide isomerase family A member 3 (PDIA3), and heat shock proteins (HSPs), the active secretion of metabolites and cytokines, such as adenosine triphosphate (ATP), type I interferon (IFN-I), C-X-C motif chemokine ligand 9 (CXCL9), CXCL10, interleukin-1β (IL-1β), and IL-6, and the passive release of soluble macromolecules, such as the cytosolic protein annexin A1 (ANXA1), the nuclear chromatin-binding protein high mobility group box 1 (HMGB1), DNA, and RNA. These changes occur in the surface components and secretions of stressed or dying cancer cells, and the hidden “danger signals” within the cells are referred to as damage-associated molecular patterns (DAMPs). DAMPs are a kind of endogenous dangerous molecules released into the intercellular space or blood circulation after the tissue or cells are stimulated by injury, hypoxia, stress and other factors. DAMPs can activate the innate immune system by interacting with receptors, thus initiating the immune response (13). These “danger signals” are released from the cells to recruit immune cells and activate the immune response (14, 15). CALR is mainly exposed on the cell membrane of tumor cells as an “eat me” signal to promote its uptake by dendritic cells (DCs) and other phagocytes, thereby activating the innate and adaptive immune responses (14). In the process of ICD, ATP is released in an autophagy-dependent manner. Through exocytosis, ATP-containing vesicles are released from the cells as a “find me” signal to recruit macrophages and DCs to the local tumor and induce DC maturation (16). ANXA1 is an important factor for DC homing. HMGB1 can bind to the relevant receptors on the DC membrane, transmit signals to mature DCs, and present antigens to cytotoxic T lymphocytes (CTLs), activating relevant signaling pathways and cellular immunity (17). The RNA and DNA passively released during the ICD process can stimulate IFN-I, and then bind to the receptors on the surface of the immune cells to activate the body’s immunity. The main role of IFNs in ICD is to activate signal cascades, which produce more effective IFNs by autocrine and paracrine mechanisms, and then activate the innate and adaptive immune systems. When exposed on the cell surface or released from the cell, these components can recruit immune cells, induce inflammatory responses, stimulate the release of immune factors, and ultimately eliminate tumors via their immunosuppressive function (18) (**Figure 1**).

## 2 Classification of ICD inducers

### 2.1 ICD induced by drugs

#### 2.1.1 Anthracycline conventional chemotherapy drugs

Anthracycline conventional drugs are important anti-tumor chemotherapeutic agents that primarily exert their effects through DNA cross-linking, thereby interfering with DNA replication and transcription, and inhibiting the proliferation of cancer cells. Examples such as doxorubicin (DOX), mitoxantrone (MTX), and epirubicin (EPI), can induce ICD (19–21). These anthracycline conventional chemotherapy drugs can trigger endoplasmic reticulum (ER) stress, leading to rapid phosphorylation of eukaryotic translation initiation factor 2α (eIF2α). This subsequently results in the exposure of CALR, ATP, and HMGB1, and the release of HSP70 and HSP90. After translocation to the cell surface, these markers present signals to CD8^+^ T cells, induce natural killer cell activation, and promote DC maturation, thus triggering an anti-tumor immune response and inducing the ICD of tumor cells (19, 22, 23). DOX, an anthracycline antibiotic, has been used to treat cancer for more than 40 years and is one of the most effective anti-cancer drugs (24). Tumor microenvironment is the surrounding environment for the survival and development of tumor cells, which plays an important role in tumor growth, metastasis and response to treatment. Cancer cells can evade antibiotic activity by expressing indoleamine 2,3-dioxygenase-1 (IDO1) in the tumor microenvironment. Kim et al. prepared cancer-targeting nanoliposomes containing ICD inducers (DOX and the siRNA of IDO1) and specifically delivered these nanoliposomes to breast cancer cells through endocytosis to enhance ICD and inhibit the expression of IDO1, which shows significant toxicity in breast cancer cells, to achieve synergistic immunotherapy through tumor-specific immune regulation (25). MTX, as a second-line chemotherapy drug for prostate cancer, breast cancer, leukemia, and other cancers, can also effectively induce ICD. In prostate cancer cells, MTX upregulates the expression of protein kinase RNA-like endoplasmic reticulum kinase (PERK)/GCN2, which subsequently activates the phosphorylation of eIF2α at the S51 site, promoting the exposure of CALR on the cell surface and the release of ATP and HMGB1 extracellularly, thereby inducing ICD to enhance anti-tumor immunity *in vivo* (26). The same phenomenon was observed by Zhang et al. in the ICD of breast cancer cells induced by EPI. To better exert the role of EPI in inducing breast cancer ICD, Zhang et al. used dasatinib (DAS) to affect the function of cancer-associated fibroblasts to remodel the dense extracellular matrix. This enhanced the ability of EPI to penetrate tumor tissue through vesicles, while enhancing tumor killing, immune activation, and ultimately the therapeutic effect against breast cancer (27).

As mentioned above, anthracycline conventional chemotherapy drug-induced ICD is an effective tumor treatment strategy. Targeted intervention of the tumor microenvironment via such drugs can enhance the therapeutic effect on the tumor. In future research, we need to further explore the complexity and dynamics of the tumor microenvironment, and continue to explore the mechanisms and effects of anthracycline conventional chemotherapy drug-induced ICD, to provide more effective means of tumor treatment.

#### 2.1.2 Platinum-based chemotherapy drugs

The anti-cancer mechanism of platinum-based chemotherapeutic drugs is different from traditional anti-cancer drugs. Through extensive research and trials, the initial determination of the anti-cancer mechanism of platinum-based anti-cancer drugs has been divided into four steps: trans-membrane transport, hydration dissociation, targeted migration, and activity toward DNA, which causes DNA replication disorders and thus inhibits the division of cancer cells (28). Oxaliplatin (OXP) is the first platinum-based ICD inducer that has been applied in the treatment of colorectal cancer, hepatocellular carcinoma, and gastric cancer. OXP binds to double-stranded DNA and releases tumor-related antigens, promoting the “eat me” signal, and thereby promoting ICD (29). Gu et al. developed liposomes containing OXP and combined them with the stimulator of interferon genes (STING) agonist (ADU-S100) for the treatment of colorectal cancer. The liposomes effectively inhibited the proliferation of tumor cells, while inducing ICD of CT26 colorectal cancer cells, and enhanced the maturation and phagocytic ability of dendritic cells *in vitro* (30). Zhu et al. found that when OXP was used to treat human and mouse hepatocellular carcinoma cells, the biomarkers related to ICD were significantly enhanced. In addition, when immature DCs were co cultured with OXP-treated H22 cells, the number of mature DCs increased. The *in vitro* and *in vivo* data showed that OXP could induce ICD of hepatocellular carcinoma cells of different species and subsequently generate anti-tumor immune responses (31). Cisplatin is considered to be a suboptimal ICD inducer because it cannot directly induce CALR exposure on the cell surface, and its effect is far inferior to OXP (32). Therefore, a better understanding of the determinants of ICD induced by chemotherapeutic drugs will help to transform relatively weak ICD inducers into strong ICD inducers to develop more effective anti-cancer strategies. Yang et al. found that the combination of IFN-β and cisplatin can enhance the expression of ICD biomarkers in cancer cells, including the cell surface exposure of ER chaperones such as CALR, ERp57, and HSP70/90, and the phosphorylation of eIF2α. These results indicate that exogenous IFN-β may be the decisive factor for the conversion of cisplatin to an ICD inducer, ultimately meaning that the combination of IFN-β with cisplatin becomes a more effective anti-cancer regimen (33).

At present, platinum inducers are not extensively used in clinical treatment. Carboplatin and nedaplatin, second-generation platinum-based chemotherapy drugs, differ from cisplatin in their ability to induce ICD because of their distinct mechanisms of action and biological effects. Carboplatin and nedaplatin mainly affect the transcription and replication of DNA through the formation of Pt-DNA adducts, leading to the death of tumor cells. This process mainly involves the structural deformation of DNA, rather than directly inducing ICD. In addition, although carboplatin and nedaplatin can effectively inhibit the growth of tumor cells, they are unable to effectively release signal molecules, such as CALR, ATP, and HMGB1, due to their lack of a direct ICD induction mechanism, so they fail to induce the ICD of tumor cells (34). Metal ICD inducers have shown great potential in cancer immunotherapy, but the effect of their structure on DNA and their mechanism of inducing ICD still need to be actively explored in basic and clinical research.

#### 2.1.3 Targeted drugs

Inducing ICD in tumor cells is not limited to traditional chemotherapeutic drugs, and some targeted drugs can also play a role in anti-tumor immunity. Targeted drugs can prevent the growth and proliferation of cancer cells by identifying and interacting with key sites on tumor cell-specific genes. Some evidence exists that targeted drugs can also induce ICD in tumor cells. Cetuximab, an epidermal growth factor receptor (EGFR) antagonist, can induce ICD. Cetuximab can promote the migration of CALR and ER-related protein disulfide isomerase ERp57 from the ER lumen to the cell membrane of dying cells prior to cell death, and cause the phosphorylation of translation initiation factor eIF2α (35). Pozzi et al. found that cetuximab alone or in combination with FOLFIRI (folinic acid, bolus/continuous fluorouracil, and irinotecan) can induce the translocation of CALR and ERp57 to the cell membrane, but FOLFIRI alone failed to stimulate ICD. Quantitative proteomic mass spectrometry analysis confirmed that cetuximab promoted the ER stress response and the translocation of ER-related protein to the plasma membrane, which induced ICD, while enabling DCs to engulf dead tumor cells, leading to a protective CD8^+^ T memory cell immune response (36). Anti-EGFR-specific antibody 7A7 also exerts similar effects. In a study of D122 mouse lung cancer cells, the clinical efficacy of anti-EGFR-specific antibody 7A7 treatment was consistent with that of anthracycline conventional chemotherapy drug-induced ICD, which can significantly induce CALR on the plasma membrane to be translocated to the cell surface. 7A7 is conducive to stimulating DC maturation and increasing the infiltration of CD4^+^ T cells and CD8^+^ T cells (37). Furthermore, as a multi-target tyrosine kinase inhibitor (ALK/ROS1/MET), crizotinib can induce ICD in both human and mouse cells. Liu et al. found that human U2OS, HeLa, and HCT-116 cell lines and mouse fibrosarcoma MCA205 cells treated with tyrosine kinase inhibitors, such as crizotinib, could cause CALR exposure on the cell surface and the release of ATP and HMGB1, indicating that crizotinib has potential as an ICD inducer (38).

Targeted drugs can promote the occurrence of ICD by inhibiting the growth and proliferation of tumor cells. This cell death can trigger the body’s immune response and enhance anti-tumor immunity. In addition, targeted drugs can also affect the tumor microenvironment, change the immune phenotype of tumor cells, and make them more recognizable by the immune system. In conclusion, the induction of ICD by targeted drugs is now considered a potential immunotherapy for cancer. However, the specific mechanism involved in this process requires further study. Understanding its mechanism of action and influencing factors will help to make better use of targeted drugs for anti-tumor treatment. At the same time, the possible adverse reactions and side effects also need to be closely monitored and evaluated (**Table 1**).

**Table 1:** Examples of drugs as immunogenic cell death inducers.

Class	Agent	Observations	Ref.
Anthracycline conventional chemotherapy drugs	DOX	DOX and IDO1-siRNA form nanoliposomes, which can target tumor cells through endocytosis, enhance ICD effect and inhibit IDO1 expression	(24, 25)
	MTX	MTX promotes the release of DAMPs by up regulating the expression of PERK/GCN2 and activating the phosphorylation of S51 site of eIF2α	(26)
	EPI	DAS promotes the ability of EPI to penetrate into tumor tissues through vesicles, promotes immune activation, and induces the release of DAMPs	(27)
Platinum-based chemotherapy drugs	OXP	OXP releases tumor associated antigens through a large number of dsDNA crosslinks and promotes the maturation of DCs to promote the occurrence of ICD	(29–31)
	Cisplatin	IFN-β combined with cisplatin can enhance the exposure of ICD markers CALR, ERp57 and HSP70/90 and the phosphorylation of eIF2α	(32, 33)
Targeted drugs	Cetuximab	Cetuximab translocates CALR and ERp57 from the ER lumen to the cell membrane of dying cells and causes the phosphorylation of eIF2α	(35, 36)
	7A7	The clinical efficacy of 7A7 treatment was consistent with anthracycline conventional chemotherapy drugs induced ICD	(37)
	Crizotinib	Treatment with tyrosine kinase inhibitors such as crizotinib leads to CALR exposure, ATP and HMGB1 release	(38)

ICD, immunogenic cell death; CALR, calreticulin; DOX, doxorubicin; IDO1, indoleamine 2,3-dioxygenase-1; MTX, mitoxantrone; EPI, epirubicin; DAS, dasatinib; DAMPs, damage-associated molecular patterns; OXP, oxaliplatin; DCs, dendritic cells; dsDNA, double-stranded DNA; HMGB1, high mobility group box-1; ATP, triphosphate.

#### 2.1.4 Antibody-drug conjugates

In recent years, antibody-drug conjugates (ADCs) have not only played a significant role in tumor treatment but have also demonstrated great potential in inducing ICD in tumor cells. ADCs are large macromolecular complexes based on antibodies, predominantly composed of three major components: antibodies that selectively recognize tumor cell surface antigens, cytotoxic drugs that kill tumor cells, and linkers that connect the antibodies and drugs (39). After ADCs bind to the surface antigens of target cells through antibodies, they are internalized through endocytosis, allowing the drugs to enter tumor cells. Then, the linkers break under specific conditions within the tumor cells, releasing highly active cytotoxic drugs, thereby killing the tumor cells. In this way, the drugs specifically target tumor cells.

Trastuzumab emtansine (T-DM1) is the first approved ADC for difficult-to-treat HER2^+^ breast cancer. Although it shows significant efficacy in the early clinical stage, drug resistance is relatively common. Mustafa Emre Gedik et al. found that in sensitive cells, T-DM1 induces spindle assembly checkpoint-dependent ICD by inducing eIF2α phosphorylation, CALR surface exposure, ATP and HMGB1 release, and secretion of ICD-related cytokines. However, these characteristics are lost in T-DM1-resistant cells. In resistant cells, transforming acidic coiled-coil containing 3 (TACC3) is overexpressed, and the inhibition of TACC3 can restore T-DM1-induced ICD (40). In summary, these results indicated that ICD is the key mechanism of T-DM1, and targeting TACC3 in resistant cells can restore T-DM1-mediated ICD. Additionally, ADC therapy has shown great potential in the treatment of triple-negative breast cancer (TNBC). However, the current specific ADC for TNBC, sacituzumab govitecan, was unable to overcome the inhibitory effect of adenosine in the immune microenvironment. Xiao Xie et al. therefore proposed a new ADC, a dual immunostimulatory complex αCD73-PLG-MMAE, which can specifically target TNBC and regulate tumor immunity through mechanisms such as ICD and interference with the adenosine signaling pathway. This ADC kills tumor cells through cytotoxic drugs, comprehensively regulates immunosuppression, and restores the body’s persistent immune response (41). Their study presented an antibody-polymer drug complex with immunomodulatory and immune agonist effects, providing new insights into the treatment of TNBC. Guillem Pascual-Posto et al. found that D3-GPC2-PBD, an ADC carrying a pyrrolobenzodiazepine (PBD) dimer, can target the highly differentially expressed cell surface oncogene glypican 2 (GPC2) in neuroblastoma. This ADC can induce ICD in neuroblastoma cells, characterized by CALR and HSP70/90 translocation, as well as HMGB1 and ATP release, and promote tumor phagocytosis by macrophages (42). Based on this finding, ADC combined immunotherapy has demonstrated strong efficacy in various neuroblastoma clinical models.

In summary, ADCs and ICD have significant potential applications in cancer treatment. The precise targeting ability and high-activity drug release capacity of ADCs mean that they have broad application prospects in cancer therapy, especially for tumor types that are insensitive to traditional chemotherapy drugs or prone to drug resistance. In addition, through in-depth research on the regulatory mechanisms of ICD induced by ADCs, combined treatment regimens may be further optimized. This is expected to provide more effective and long-lasting treatment options for cancer patients, improving treatment outcomes and patient survival rates. In the future, with continuous technological progress and in-depth research, the study of ADCs and ICD will further promote the advancement and development of cancer treatments.

### 2.2 ICD induced by oncolytic viruses

Oncolytic viruses are a class of virus that have the ability to replicate and kill cancer cells, and the use of oncolytic viruses for cancer treatment has become a promising new therapy (43). It has been reported that a variety of oncolytic viruses can induce ICD of tumor cells, and these include Newcastle disease virus (NDV), measles virus (MV), herpes simplex virus type 1 (HSV-1), and parvovirus H1 (H-1PV), which has laid a theoretical foundation for the combination of oncolytic viruses with immunotherapy (44, 45). NDV is a negative-strand RNA virus that can specifically infect tumor cells. It continuously proliferates and replicates in tumor cells to kill and degrade tumor cells. The viruses released from degraded cells can further infect surrounding tumor cells, but have little impact on normal tissue and cells (46). NDV can not only directly degrade tumors, but can also cause ICD (47). Ye et al. found that oncolytic NDV, strain FMW (NDV/FMW) triggers the exposure of CALR on the cell surface and the release of HMGB1 and HSP70/90 in lung cancer cells *in vitro*. In the xenotransplantation model, the supernatant from NDV-infected cells reduced tumor formation, indicating that NDV-induced cancer cell death is immunogenic and related to the release of major ICD determinants. In summary, these results show that oncolytic NDV is an effective ICD inducer (48).

MV is a single-stranded RNA paramyxovirus, which is a common type of oncolytic virus. Studies have shown that MV can cause the ICD of human melanoma cells. After infection with MV, the levels of inflammatory cytokines IL-6 and IL-8 released by cells increase in a dose-dependent manner. In addition, melanoma cell lines killed by MV can secrete HMGB1, IFN-α, IFN-β, and IFN-λ to varying degrees, thereby triggering the host’s innate and adaptive anti-tumor immune responses and inducing the ICD of melanoma cells (49). HSV-1 is a double-stranded DNA neurotropic virus with high infection efficiency, which can easily be genetically modified. To date, HSV-1 has been widely studied as an oncolytic virus in basic and clinical research (50). Takasu et al. found that HSV-1 can also cause ICD in squamous cell carcinoma (SCC) cells. When mouse SCCVII cells are infected with HSV-1, DAMPs are produced in SCC cells to induce cell death. At the same time, ATP and HMGB1 are released from the cells, and CALR is transferred to the cell membrane. In C3H mice, the growth of SCCVII tumors infected with HSV-1 is significantly less than that of the control group, which indicates that oncolytic HSV-1 can cause ICD of tumor cells (51). Inducing tumor cell ICD is often used as a treatment strategy for highly invasive malignant tumors. Pancreatic ductal adenocarcinoma (PDAC) is a highly invasive disease and it has been proposed that forcing the ICD of such tumor cells is an effective method to trigger an adaptive immune response to this cancer (52, 53). Oncolytic H-1PV is a DNA virus that can participate in the ICD of PDAC cells and is effective at the clearance of these tumor cells. H-1PV can activate a variety of cell death pathways and increase the release of extracellular HMGB1 by 4.0 ± 0.5 times. In addition to its own oncolytic effect, it may also convert gemcitabine-induced apoptosis of PDAC cells into ICD. The compatibility of H-1PV with other ICD inducers is conducive to the incorporation of H-1PV into multimodal anti-cancer therapies (54). In addition, Zika virus and Seneca Valley virus, both of which have oncolytic activity, may also be involved in the progression of ICD (55–57). Zika virus infection has been proven to induce ER stress by triggering the unfolded protein response, and cause oxidative stress and redox imbalance. ER stress is precisely the key molecular mechanism of ICD. Whether the engineered Zika virus can induce tumor ICD by affecting ER stress and ROS expression is worth exploring for its further application (58). Similarly, Seneca Valley Virus, which has anti-tumor effects, has entered phase II clinical trials in tumor treatment, but the mechanism by which it induces ICD deserves further exploration (57).

Viruses can be divided into two categories according to their genetic material: DNA viruses and RNA viruses. Both types of viruses may cause ICD during infection. In the process of infection, DNA viruses can indirectly cause ICD by affecting the signal transduction pathways of the host. Compared with DNA viruses, the genetic material of RNA viruses is more unstable. This instability may make RNA viruses more prone to trigger strong immune responses and cell death. RNA viruses often produce a large number of harmful proteins and RNA fragments in host cells during the process of replication. These substances may activate the defense mechanism of cells and lead to ICD. In summary, ICD induced by oncolytic viruses is a complex and important research field. In the future, we hope to gain a better understanding of the pathogenic mechanisms and interactions between viruses and host cells through more in-depth research, which will provide a theoretical basis for the development of new antiviral drugs and treatment strategies (**Table 2**).

**Table 2:** Examples of oncolytic virus available immunogenic cell death inducers.

Class	Agent	Genetic material	Observations	Ref.
Oncolytic virus	NDV	A negative strand RNA virus	NDV can specifically infect tumor cells, trigger CALR exposure and the release of HMGB1 and HSP70/90	(47, 48)
	MV	A single stranded RNA paramyxovirus	MV can cause the increase of inflammatory cytokines IL6, IL8, IFN-α, IFN-β and IFN-λ, as well as the release of HMGB1	(49)
	HSV-1	A double stranded DNA neurotropic virus	HSV-1 can trigger CALR exposure and the release of HMGB1 and ATP	(50, 51)
	H-1PV	A single stranded DNA virus	H-1PV can increase the release of HMGB1 by 4.0 ± 0.5 times, inducing the occurrence of ICD	(54)

NDV, Newcastle disease virus; MV, measles virus; HSV-1, herpes simplex virus type 1; H-1PV, parvovirus H1; CALR: calreticulin; IL-6, Interleukin-6; IL-8, Interleukin-8; IFN-α, interferon-α; IFN-β, interferon-β; IFN-λ, interferon-λ; HMGB1, high mobility group box-1; ATP, adenosine triphosphate; ICD, immunogenic cell death.

### 2.3 ICD induced by epigenetic modifiers

Epigenetic modifications regulate the transcriptional activity of genes and protein expression without altering the DNA sequence (59, 60). DNA methyltransferase, histone deacetylase (HDACs), proteasome inhibitors, and other epigenetic modulators can enhance the immune response by increasing the expression of antigens, co-stimulatory molecules, and major histocompatibility complex (MHC) molecules. Epigenetic modifiers fundamentally change the immunogenicity of tumor cells while affecting the tumor microenvironment, interfere with immune evasion of tumor cells, and finally play an role in the anti-tumor immune response (61). There is also a certain connection between epigenetic modifications and ICD. Epigenetic modifiers, as ICD inducers, actively participate in the regulation of ICD-related DAMP release, further improving the efficacy of cancer immunotherapy (62). Therefore, epigenetic modifiers also play an important regulatory role in anti-tumor immunity.

**Figure 2:**
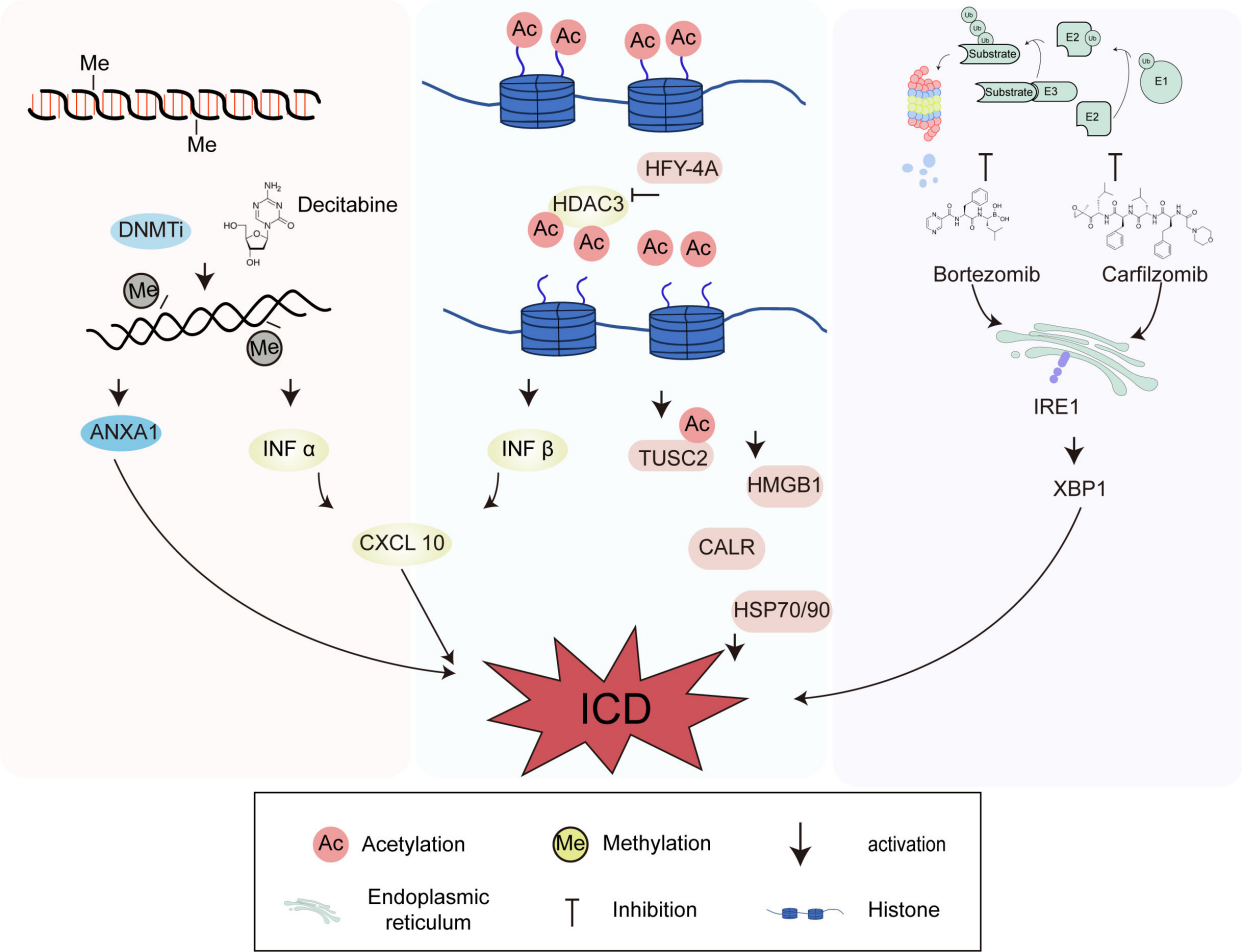
Immunogenic cell death (ICD) induced by epigenetic modifiers of DNA methyltransferase, histone deacetylase and proteasome inhibitors. DNA methyltransferase, histone deacetylase, and proteasome inhibitors can enhance immune responses and ultimately regulate ICD. The histone deacetylase HDAC3 and the demethylating agent decitabine can promote interferon-I signaling and increase CXCL10 expression, which further induce ICD. HFY-4A can promote the transcription of TUSC2 by facilitating the acetylation of histone H3K56 on the TUSC2 promoter. The expression of ANXA1 is inhibited by methylation, and DNMTi can restore the expression and secretion of ANXA1 during ICD. Bortezomib and carfilzomib can induce the expression of CRT through the endoplasmic reticulum stress pathway IRE1-XBP1.

The main role of IFN-I in ICD is to activate the signal cascade, attract antigen-presenting cells (APCs) to the tumor microenvironment, and play a key role in the maturation of APCs and the activation of T cells (63, 64). There is also a clear connection between epigenetic modifications and IFN regulation. First, IFN-I (IFN-α and IFN-β) mainly signals through the heterodimeric IFN-α receptor to trigger a wide range of responses. It has been found that the expression of histone deacetylase HDAC3 is necessary for IFN-β expression, indicating that HDAC3 plays a regulatory role in controlling IFN-β expression (65). Second, CXCL10 secretion is a result of IFN signaling. In ovarian cancer, treatment with the demethylating agent decitabine can promote IFN-I signaling and increase CXCL10 expression, which further induces ICD with therapeutic effects (66, 67). Additionally, the novel HDAC inhibitor HFY-4A can dose-dependently induce ICD in breast cancer cells, characterized by high expression of HMGB1, CALR, HSP70, and HSP90. Tumor suppressor candidate 2 (TUSC2) is a candidate tumor suppressor gene located on the short arm of human chromosome 3p21.3 that is widely involved in processes such as gene transcription, cell cycle progression, and apoptosis. HFY-4A can promote the transcription of TUSC2 by facilitating the acetylation of histone H3K56 on the TUSC2 promoter, enhancing ICD in breast cancer cells (68). ANXA1 is one of the DAMPs released by the immune system during ICD and is also regulated by epigenetic modifications. In nasopharyngeal carcinoma cell lines, the expression of ANXA1 is inhibited by methylation, and DNA methyltransferase inhibitors can restore the expression and secretion of ANXA1 during ICD (69). Matsushita et al. found that the proteasome inhibitors bortezomib and carfilzomib can induce ICD in myeloma cells. Even at low concentrations, bortezomib and carfilzomib can induce the expression of CALR on the surface of myeloma cell lines and in some patient samples through the ER stress pathway IRE1-XBP1, and cause myeloma cells to secrete large amounts of HMGB1, subsequently promoting the activation of DCs. Proteasome inhibitors-induced ICD is expected to improve the prognosis of multiple myeloma patients (70). As mentioned above, there is an undeniable connection between the regulation of ICD markers and epigenetics. Understanding the regulatory role of epigenetic modifiers in ICD may provide a new approach for improving the efficacy of ICD and current cancer immunotherapy (**Figure 2**).

### 2.4 ICD induced by physical stimulation

As an effective means to enhance the immunogenicity of tumor cells and activate the immune response, physical stimulation has been widely studied and applied in clinical practice (71). Radiotherapy (RT), photosensitizers (PSs) for photodynamic therapy (PDT), photothermal therapy (PTT), nano-pulse therapy, and other physical stimuli can induce the occurrence of ICD in the tumor microenvironment (72).

#### 2.4.1 Radiosensitizers for radiotherapy

In clinical applications, it has been found that RT mainly damages the DNA double strands and the chemical bonds between the bases of various components within cells, stimulates the anti-tumor effect mediated by T cells, induces DNA damage and tumor cell apoptosis, and ultimately kills tumor cells (73–75). ICD can also be induced during RT, which is accompanied by the translocation and exposure of CALR on the cell surface and the release of DAMPs such as HMGB1 and ATP. These molecules act as “danger signals” and can activate inflammatory pathways and innate immune responses, promoting the activation and migration of immune cells (76). Additionally, RT can induce the secretion of pro-inflammatory cytokines and chemokines, such as IFN-I, IL-1, tumor necrosis factor (TNF), and CXCL16, to stimulate DC maturation and promote the anti-tumor effect of CD8^+^ T cells (77). Clinically, RT induces ICD of tumor cells in a dose-dependent manner, and typically 2–20 Gy can effectively induce ICD (78). It is reported that nano metal organic frameworks are excellent radiosensitizers that can load drugs or macromolecules into their porous structures with high efficiency and excellent biocompatibility, and have been widely applied in RT. The IDO inhibitor INCB024360 in the core and channels of nano metal organic frameworks can significantly increase the number of tumor-infiltrating cytotoxic CD8^+^ T cells and further induce ICD at a lower dose of X-ray irradiation (79, 80). The combination of radiosensitizers with RT minimizes the side effects of RT while improving the cure rates of primary and distant tumors.

#### 2.4.2 Photosensitizer-mediated photodynamic therapy

PDT is a minimally invasive treatment method in which a PS generates an oxidative stress response through light activation at a specific wavelength, leading to the destruction of tumor cells at the site of PS activity, thereby specifically targeting abnormal cells (81). According to their chemical structure, PSs can be divided into porphyrin and non-porphyrin compounds. The first clinically approved PSs were hematoporphyrin derivatives, which are still used in clinic, such as for the treatment of cervical cancer (82), esophageal cancer (83), and colorectal cancer (84). The most common non-porphyrin PSs include hypericin and hypocrellin. To further improve the pharmacokinetics of PSs and reduce their side effects, a new PS combining a photoactive chromophore with targeted drugs or carriers is currently being studied. These combination PSs can be specifically delivered to cancer cells for targeted and selective killing (81). PDT can trigger ER stress by changing ER homeostasis, selectively targeting the ER and releasing more DAMPs, while further inducing ICD in tumor cells (71, 85).

As early as 2004, first-generation porphyrin administration was reported to cause extensive exposure of HSPs on the surface of mouse colon cancer cells, which were subsequently phagocytosed by DCs, accompanied by the production of IL-12, thereby inducing the ICD of mouse colon cancer cells (86). Additionally, Korbelik et al. examined the expression of CALR and HMGB1 in Lewis lung cancer (LLC) cells and the LLC tumor tissue of mice after PDT. They found that the expression of CALR on the cell membrane was considerably increased both *in vitro* and *in vivo* one hour after PDT. At the same time, an increase in HMGB1 was detected in the serum of host mice (87). These data suggest that PDT with porphyrin as the PS is a potent inducer of ICD. PDT mediated by PSs, such as 2-[1-hexyloxyethyl]-2-devinyl pyropheophorbide-α (HPPH) and hypericin, can also induce ICD under specific conditions. To maximize the ICD effect, Yang et al. constructed a pH-responsive block copolymer of polyethylene glycol-b-cationic polypeptide (PEG-b-cPPT), which not only formed self-assembled nanovesicles but also served as an inducer of ICD. The photosensitizer HPPH and the IDO inhibitor indoximod (IND) were encapsulated in the nanovesicles by hydrophobic interactions. HPPH and IND were finally transported to the cytoplasm through vesicles (88), which could directly kill tumor cells and induce ICD while generating reactive oxygen species (ROS). The nanovesicles can not only be used as a carrier to deliver HPPH and IND, but also induce the transfer of CALR from the ER membrane to the cell membrane surface, thus recruiting DCs into tumor tissues and providing additional immune stimulation to activate an immune response. Therefore, this nanovesicle delivery system has been proven to exert a strong anti-tumor effect. In addition, hypericin, a well-studied PS in this field, is capable of inducing targeted ICD, and can be targeted and localized in the ER. After irradiation, it localizes to the ER, causes ER stress, and activates related signaling pathways. Hypericin-mediated PDT can also inhibit the activity of nuclear factor kappa-B (NF-κB) in tumor cells, downregulate tumor-promoting cytokines derived from tumor cells, and ultimately lead to ICD (89, 90).

The selection of PSs is a key issue in PDT. At present, many PSs are being developed and studied. A mixture of porphyrin and its derivatives as PSs for PDT are limited in their clinical application by their need to be protected from light for 1–2 months after administration. Newly developed non-porphyrin PSs, such as hypericin and combined PSs, offer the advantages of a clear chemical structure, stable properties, fast absorption and metabolism in the body, a short photophobic time, and strong PS activity. ICD induced by PSs has gradually become a potential treatment method, and ICD-induced PDT has broad research prospects.

#### 2.4.3 Photothermal therapy

PTT is a novel approach for tumor treatment with great potential for development. This therapy uses materials with high photothermal conversion efficiency to convert light energy into heat energy under the irradiation of an external light source, thereby generating high temperatures in tumor tissues through photothermal converters in a minimally invasive manner to kill tumor cells (91, 92). PTT can also trigger the occurrence of ICD. When the temperature rises to 41°C–48°C, the DNA within tumor cells is damaged and proteins denature, leading to the release of tumor-associated antigens and ICD-related HSPs. After recognizing this “danger signal,” APCs present these signals to CD8^+^ T cells, thereby activating adaptive immunity (93). However, because of the shallow penetration depth of the light source in the tissue and the poor immune activation effect, PTT alone cannot effectively ablate a distal metastatic tumor. Therefore, photothermal converters are often combined with immunotherapeutic agents, such as anti-PD-1/PD-L1 antibodies, IDO inhibitors, and immune adjuvants, to enhance immune stimulation and alleviate immune suppression in the tumor microenvironment, thereby improving therapeutic efficacy (94). Zhao et al. proposed a novel two-dimensional nanomaterial black phosphorus, as a near-infrared-responsive nanophotothermal converter for PTT and electrostatically bound the immunogenic adjuvant CpG to the nanosheets to enhance the immune response. At a temperature close to 45.6°C, black phosphorus triggers programmed necroptosis, thereby initiating the ICD process. CALR is transferred to the cell membrane, while HMGB1 is released and ATP is secreted, which promoted antigen presentation and the secretion of cytokines such as TNF-α, IFN-α, and IL-2 (95). Prussian blue can also serve as a near-infrared-absorbing photothermal converter in PTT. Loading sorafenib onto Prussian blue nanoparticles and conjugating them with the SP94-targeting peptide can be used to treat hepatocellular carcinoma. Under 808 nm laser irradiation, the temperature of liver cancer tissue rises from 36.2°C to 49.2°C, leading to the cell surface exposure and release of tumor-associated antigens, CALR, ATP, and HMGB1, inducing the occurrence of ICD. Prussian blue nanoparticles alleviate the hypoxic tumor microenvironment, reduce the expression of hypoxia-inducible factor-alpha and the number of M2 macrophages, while enhancing the maturation of DCs and the infiltration of CTLs (96). PTT based on black phosphorus and Prussian blue exhibits significant anti-tumor effects, resulting in a significant reduction in tumor growth rate. The combination of PTT and ICD immunotherapy is gradually becoming a promising treatment approach.

#### 2.4.4 Nano-pulse stimulation

Nano-pulse stimulation (NPS) is a novel non-thermal and drug-free bioelectric therapy. This therapy involves ultrashort electrical pulse stimulation of tumor cells, triggering regulated cell death, eliminating tumors, and inhibiting the growth of secondary tumors (97, 98). The other physical methods mentioned above have been proven to effectively trigger anti-tumor immunity through the induction of ICD. As a physical method that causes cell death, NPS has been shown to induce the occurrence of ICD in cells and cause the classic indicators of ICD, such as the transfer of CALR from the ER to the plasma membrane, the secretion of ATP, and the release of HMGB1, with levels comparable to those of the known ICD inducers doxorubicin and mitoxantrone, two anthracycline chemotherapy drugs (99, 100). Nuccitelli et al. clearly demonstrated that NPS is a physical method of therapy that, when delivered with specific energy, can induce apoptosis in tumor cells while also triggering ICD. That is, NPS can not only stimulate the activation of caspase 3/7, but also release three key DAMPs: CALR, ATP, and HMGB1. When NPS is within the energy range of 15 to 50 J/mL, the expression of CALR on the cell surface increases in an energy-dependent manner and the secretion of ATP peaks at 15 J/mL and then decreases at 25 J/mL. The release of HMGB1 increases along with NPS, and reaches a level equivalent to that of the anthracycline treatment group between 10 and 25 J/ml. NPS therapy with specific energy can trigger the expression of three key signals of ICD (100). Therefore, NPS can also be regarded as a physical form of therapy that can induce ICD in tumor cells (**Table 3**).

**Table 3:** Examples of immunogenic cell death inducers triggered by physical stimulation.

Class	Agent	Observations	Ref.
Physical stimulation	RT	ICD induced during RT is characterized by the exposure and release of DAMPs, which can also induce the secretion of pro-inflammatory cytokines and chemokines IFN-I, IL-1, TNF, and CXCL16	(76, 77)
	PDT	PDT can trigger ER stress by directly changing ER homeostasis, releasing more DAMPs while inducing ICD of tumor cells	(71, 85)
	PTT	PTT can also trigger the occurrence of ICD, leading to the release of DAMPs and the secretion of cytokines such as TNF-α, IFN-α, and IL-2	(93, 95)
	NPS	NPS can cause the classic indicators of ICD, such as the transfer of CALR from the endoplasmic reticulum to the plasma membrane, as well as the secretion of ATP and the release of HMGB1	(99, 100)

RT, radiotherapy; PDT, photodynamic therapy; PTT, photothermal therapy; NPS, Nano-pulse stimulation; ICD, Immunogenic cell death; DAMPs, damage-associated molecular patterns; IFN-1, interferon-1; IL-1, Interleukin-1; IL-2, Interleukin-2; TNF-α, tumor necrosis factor-α; IFN-α, interferon-α; CALR, calreticulin; ATP, adenosine triphosphate; HMGB1, high mobility group box-1.

## 3 ICD suppression factors

In addition to the factors inducing ICD listed above, certain factors can inhibit the occurrence of ICD. MTX and proteasome inhibitors can both effectively induce ICD in cancer cells. However, Wei et al. found that when MTX and proteasome inhibitors are used in combination, they can inhibit the occurrence of ICD (101). In prostate cancer cells, proteasome inhibitors bortezomib (BZM) and MG132 impaired MTX-induced ICD. Compared with mice treated with MTX alone, mice inoculated with RM-1 mouse prostate cancer cell lines and treated with BZM or MG132 in combination with MTX showed enhanced tumor growth and shortened overall survival. BZM or MG132 weakened MTX-induced ICD, which also indicates that MTX-induced ICD requires proteasome activation. Moreover, some pathogenic viruses can act as cytotoxicity factors and inhibit ICD by interfering with cytotoxicity and adjuvant activity (102). These pathogenic viruses usually encode and inhibit proteins such as those that promote eIF2α dephosphorylation and those that inhibit cell death. These viruses can also inhibit ICD by interfering with transcription factors such as interferon regulatory factor 3 (IRF3), limiting the expression of IFN-I receptor, or promoting the expression of CALR antagonists such as CD47 protein. In addition, these viruses can limit the antigenicity of infected cells by inhibiting the expression of MHC-I, encoding immunosuppressive factors, or inhibiting T cell effector molecules to avoid recognition by the immune system and thereby inhibit the occurrence of ICD (103). Similar to viruses, malignant tumor cells can also undergo natural selection to increase their replication potential, resist ICD, and evade immune recognition (48, 49). The blocking of ICD signaling in tumor cells, the destruction of the immune system, or changes in the cellular microenvironment can all damage the tumor-targeted immune response induced by ICD inducers. Tumor cells can inhibit the exposure of CALR on the cell surface by overexpressing V-set domain-containing T cell activation inhibitor 1 (VTCN1) (104) or endoplasmic reticulum oxidoreductase 1 alpha (ERO1A) (105). Additionally, malignant tumor cells can limit the expression of CALR by overexpressing stanniocalcin 1 (STC1), reducing CALR binding sites on the cell surface, or upregulating CD47 (106–108). Therefore, the reduction of eIF2α phosphorylation or CALR expression in tumor cells, as well as the increase in STC1 and CD47 expression, can effectively inhibit the occurrence of ICD.

Although most ICD inducers have shown substantial clinical activity, there are some obstacles to their therapeutic effects. The clinical activity of ICD inducers may be limited by inappropriate dosing and timing, inefficient combination regimens, and poor surgical treatment timing, which could directly or indirectly affect the immune adaptability of the patient. As mentioned above, some pathogenic viruses and cancer cells can disrupt ICD-related pathways to evade immune surveillance. Currently, the clinical application of ICD inducers still generally involves their combination with immunotherapy or targeted drugs for anti-tumor treatment. It is expected that in the future, personalized treatment plans can be provided for cancer patients while maximizing the therapeutic potential of ICD for various malignant tumors and reducing the factors inhibiting ICD. Future research may focus on these inhibitory factors to identify more key molecules and signaling pathways, which can serve as new targets. Based on these new targets, novel drugs may be developed to alleviate inhibition and promote ICD.

## 4 Molecular mechanisms related to ICD

### 4.1 Reactive oxygen species production and endoplasmic reticulum stress

Mitochondria and the ER play significant roles in the process of ICD (109, 110). The increase in mitochondrial outer membrane permeabilization leads to the release of cytochrome c, which in turn activates ICD. Additionally, mitochondrial oxidative phosphorylation dysfunction and the increased generation of ROS not only directly damage cellular structure and function but also induce ER stress, thereby promoting the occurrence of ICD (111–113). ER stress is a highly conserved and complex mechanism, mainly including the following three signaling pathways: the protein kinase RNA-like endoplasmic reticulum kinase (PERK)-eukaryotic initiation factor 2α (eIF2α)-ATF4 pathway, the inositol-requiring enzyme 1-α (IRE1α)-X-box protein 1 (XBP1) pathway, and the ATF6 pathway (18, 114). The generation of ROS in mitochondria and ER stress are widely involved in the regulation of ICD occurrence.

**Figure 3:**
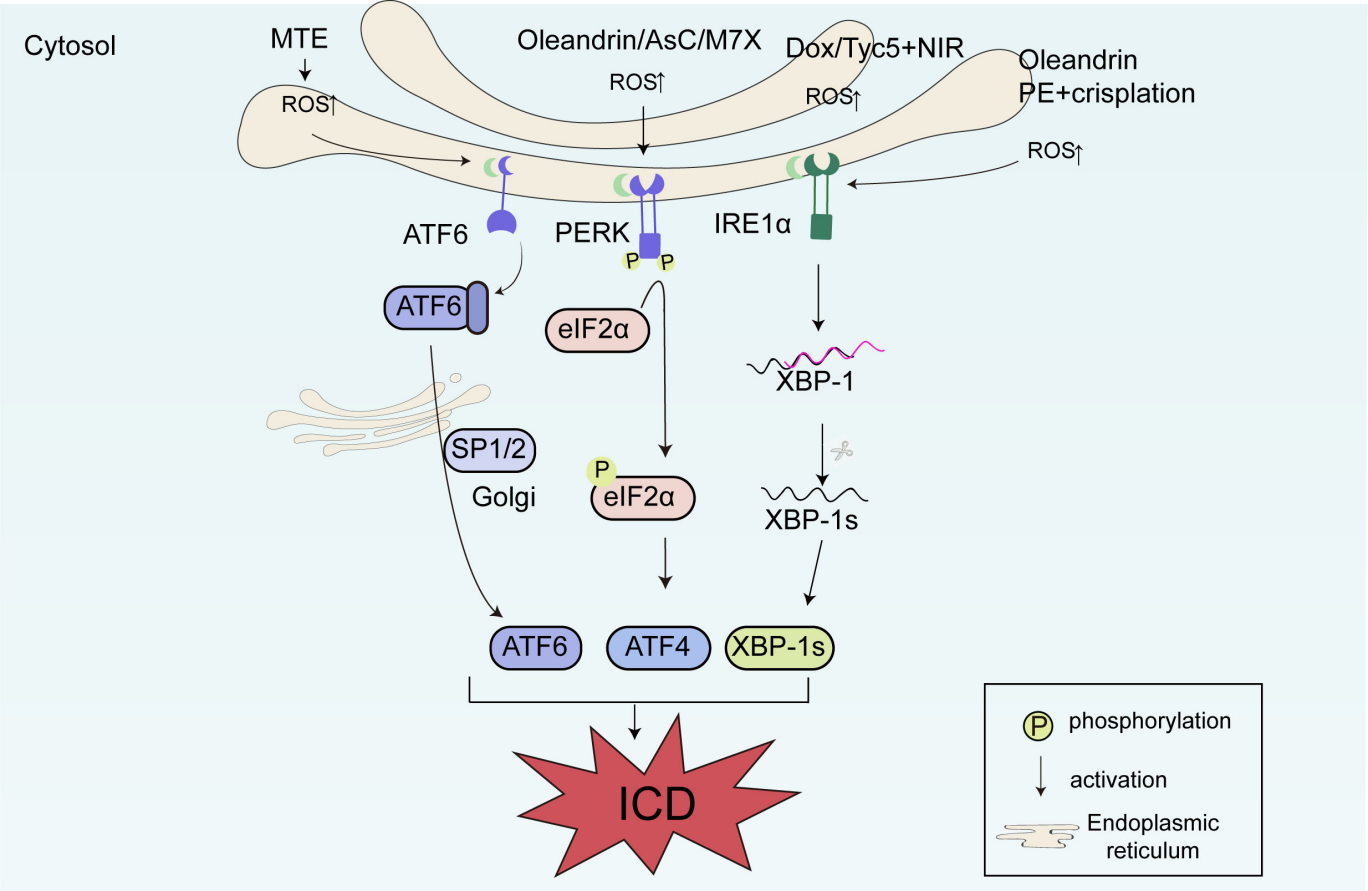
The molecular mechanisms of immunogenic cell death (ICD) related to reactive oxygen species (ROS) production and endoplasmic reticulum (ER) stress. DOX and TCy5 stimulate ICD through the large quantity of ROS produced after mitochondrial damage. Ascomylactam C (AsC) increases the formation of ROS in mitochondria, activating the PERK-eIF2α-ATF4 signaling pathway, upregulating the expression of upstream p-PERK and p-eIF2α, and downstream ATF4 and CHOP, and ultimately inducing ICD. Pinellia pedatisecta schott extract (PE) and cisplatin increase the expression of ATF3 and IRE1α. Marsdenia tenacissima extract (MTE) can induce ICD through the ATF6 pathway of ER stress. Oleandrin can simultaneously activate the PERK-eIF2α and IRE1α-XBP1 pathways to induce ICD.

The large amount of ROS produced after mitochondrial damage can widely stimulate ICD. The underlying mechanism has been further proven to be attributed to the following two pathways: (1) the increase in ROS causes ER stress, ultimately leading to the cell surface exposure of CALR, and (2) ROS promotes the release of various mitochondria-related damage molecules, including mitochondrial transcription factor A. DOX stimulates the occurrence of ICD through the large amount of ROS produced after mitochondrial damage (115). Ma et al. found that PSs can induce ER stress through the excessive production of ROS, thereby inducing ICD in breast cancer cells. Thio-pentamethine cyanine dye (TCy5) induced a large number of ROS under mild near-infrared irradiation, resulting in ER stress and an increase in DAMPs, including cell surface exposure of CALR and HSP70, and HMGB1 and ATP secretion. Experiments have shown that this inducer activates the immune response by promoting the maturation of DCs and activating CD8^+^ T cells *in vivo*, inhibiting tumor growth (116). In addition, the ER stress pathway is widely involved in ICD. Ascomylactam C (AsC) is a new 13-membered macrocyclic alkaloid. AsC has broad-spectrum cytotoxic activity and is a potential anti-tumor candidate drug. Huang et al. found that in lung cancer and melanoma cells, AsC increased the formation of ROS in mitochondria, thereby inducing ER stress, activating the PERK-eIF2α-ATF4 signaling pathway, upregulating the expression of upstream p-PERK, p-eIF2α, and downstream ATF4 and CHOP, and ultimately inducing the ICD of tumor cells (117). The PERK-eIF2α-ATF4 pathway, an important mechanism for inducing ICD, is also reported to be involved in the mechanisms of action of chemodynamic therapy and MTX (26, 118). Furthermore, p-eIF2α has been used as an important marker of ICD. Congwen et al. found that pinellia pedatisecta schott extract (PE) could induce the expression of CALR on the membrane of cervical cancer cells. When PE was used in combination with cisplatin, it more significantly induced the cell surface exposure of DAMPs such as CALR, promoting the migration of immune cells and activating DCs and CTLs. In terms of mechanism, PE and cisplatin increased the expression of upstream proteins related to ER stress, namely ATF3 and IRE1α. When the time of activity of PE was extended, the concentration of ROS in tumor cells also increased significantly. These results ultimately revealed that PE induces ER stress in tumor cells through oxidative stress, enhancing cisplatin-induced ICD in cervical cancer cells, and promoting the effect of immunochemotherapy in cancer treatment, which has important guiding significance for tumor immunotherapy (119). Yuan et al. found in non-small-cell lung cancer that marsdenia tenacissima extract (MTE) could induce ICD through the ATF6 pathway of ER stress. MTE reduced mitochondrial membrane potential, increased ROS production, and upregulated the expression of ER stress-related proteins ATF6, GRP-78, ATF4, XBP1s, and CHOP, promoting the upregulation of ICD-related markers ATP and HMGB1. *In vivo* experiments using LLC tumor-bearing mice also verified this conclusion. MTE significantly increased the expression of ER stress-related markers in tumor tissues and increased the levels of ATP and HMGB1 in the plasma of LLC tumor-bearing mice. The anti-tumor effect of MTE depends on ER stress (120). Our previous research found that oleandrin could simultaneously activate the PERK-eIF2α and IRE1α-XBP1 pathways to induce ICD (18), indicating that multiple ER stress pathways can be simultaneously activated to induce ICD (**Figure 3**).

### 4.2 Imbalance of calcium ions

**Figure 4:**
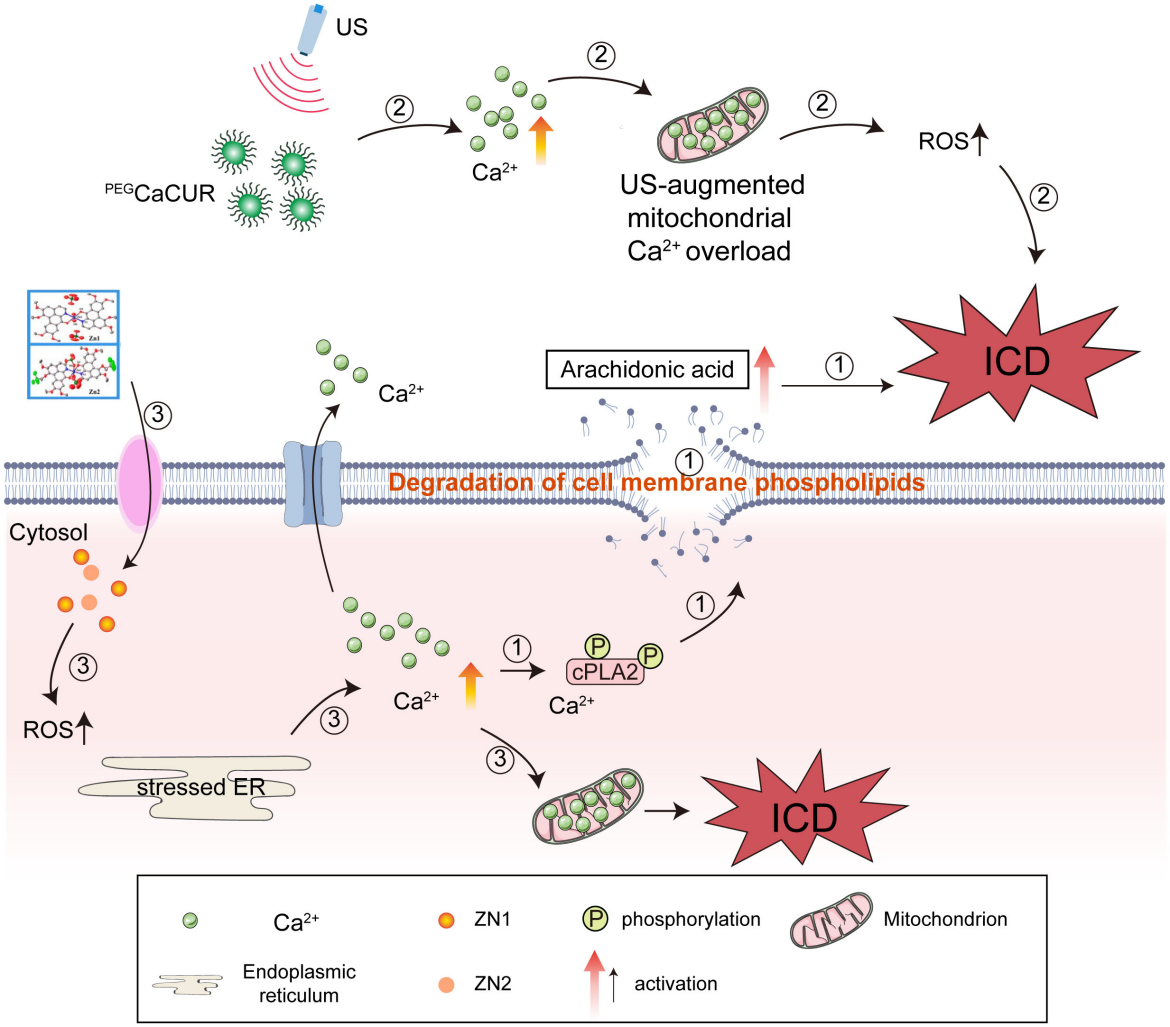
The significant impact of an imbalance in calcium ions (Ca^2+^) on immunogenic cell death (ICD). (1) An increase in calcium ion concentration activates cPLA2, which in turn leads to cell membrane damage and the activation of related pathways. (2) A calcium ion imbalance in cells mainly results from the mitochondrial calcium overload pathway. When calcium carbonate nanoparticles were incorporated with the Ca^2+^ enhancer curcumin (^PEG^CaCUR), they not only regulated mitochondrial calcium overload but also induced ICD. Moreover, when ^PEG^CaCUR were combined with ultrasound, ICD was further enhanced. (3) Zinc-based complexes Zn1 and Zn2 can activate ICD through Ca^2+^-mediated endoplasmic reticulum (ER) stress.

Calcium ions are important signaling molecules within cells, and changes in their concentration play a crucial role in the process of cell death (121). During ICD, the intracellular calcium ion concentration significantly increases, which is one of the key early events in the occurrence of ICD. The increase in calcium ion concentration activates calcium-dependent phospholipase A2 (cPLA2), which in turn leads to cell membrane damage and the activation of related pathways. The activation of cPLA2 results in the degradation of cell membrane phospholipids, generating biologically active lipid mediators such as arachidonic acid, which further promote immune responses and cell death (122). A calcium ion imbalance in cells is mainly achieved through the mitochondrial calcium overload pathway, where excessive calcium ions enter the mitochondria, causing mitochondrial dysfunction and oxidative stress, and further promoting cell death. Although many calcium ion nano regulators have been developed for treating cancer through mitochondrial Ca^2+^ overload, their ability to induce ICD remains to be explored. Zheng et al. found that when calcium carbonate nanoparticles were incorporated with the Ca^2+^ enhancer curcumin (^PEG^CaCUR), they not only regulated mitochondrial calcium overload but also induced the occurrence of ICD. Moreover, when ^PEG^CaCUR was combined with ultrasound, it further enhanced ICD, with more CALR exposed on the cell surface and increased secretion of HMGB1 and ATP to the extracellular space. This was attributed to the upregulation of ROS levels caused by enhanced mitochondrial Ca^2+^ overload, and the accumulation of ROS led to the occurrence of ICD (123). Zinc-based complexes Zn1 and Zn2 can activate ICD through Ca^2+^-mediated ER stress. Compared with Zn1, Zn2 effectively induced excessive production of ROS in the early stage, leading to ER stress. Severe ER stress caused the release of Ca^2+^ from the ER to the cytoplasm and then to the mitochondria. The imbalance of calcium ions in mitochondria leads to mitochondrial dysfunction, followed by the gradual cell surface exposure of CALR, HMGB1, and ATP-related DAMPs. Ca^2+^ overload in mitochondria provides a new design strategy for zinc-based ICD inducers (124), which could be considered to treat tumors by adjusting the concentration of calcium ions in clinic (**Figure 4**).

### 4.3 Regulation of non-coding RNA

Non-coding RNAs (ncRNAs) associated with ICD play a crucial role in tumorigenesis. Understanding the regulatory mechanism of ncRNAs on ICD is particularly important. With the in-depth study of the relationship between ncRNAs and ICD, their potential application value in inducing anti-tumor immune responses has gradually emerged. First, by regulating the expression and function of ncRNAs, the process of ICD in tumor cells can be affected, thereby enhancing the anti-tumor immune response of the body. Second, using ncRNAs as targets for immunotherapy can develop new immunotherapy methods, providing new options for tumor treatment. In addition, ncRNAs can serve as biomarkers for tumor diagnosis, providing important reference information for early diagnosis and prognosis assessment.

eIF2α is an important protein involved in the cell surface exposure of CALR. eIF2α phosphorylation is required to initiate CALR externalization. Studies have shown that ncRNA, *nc886*, can regulate eIF2α phosphorylation and thereby cause the cell surface exposure of CALR (125). Compared with normal tissue, the *nc886* gene encodes a highly methylated CpG island in cholangiocarcinoma tissue. The high methylation state of *nc886* weakens its inhibitory function on protein kinase R activation, thereby promoting eIF2α phosphorylation and CALR cell surface exposure, and positively affecting ICD induction. Conversely, high expression of *nc886* inhibits CALR exposure on the cell surface, hinders protein kinase R catalysis of eIF2α phosphorylation, and subsequently inhibits ICD in cholangiocarcinoma. Additionally, in head and neck squamous cell carcinoma, the expression of ANXA1 is negatively correlated with the expression of a specific microRNA (*miRNA-196b*). In many different cancer cell lines, the expression of *miRNA-196b* is regulated by DNA methylation, including breast cancer, colon cancer, liver cancer, and lung cancer. *miRNA-196b* directly inhibits ANXA1 expression by binding to the non-translated region of the ANXA1 mRNA transcript. When *miRNA-196b* is not expressed, ANXA1 is no longer silenced and is induced during ICD (126, 127). Similar to the regulatory mechanism of *miRNA-196b*, *miRNA-129-2* can also regulate the expression of HMGB1 through methylation. In glioma, the tumor suppressor *miRNA-129-2* can directly target HMGB1 and inhibit its release. However, when the promoter regulatory region of this miRNA is highly methylated, the expression of *miRNA-129-2* is inhibited, subsequently leading to HMGB1 expression and further triggering ICD in tumor cells (128). Shu et al. constructed a prognostic model of long ncRNA regulating ICD using data from lung adenocarcinoma patients available in the TCGA database, which is of great value in evaluating the prognosis of lung adenocarcinoma patients and guiding clinical treatment (129).

**Figure 5:**
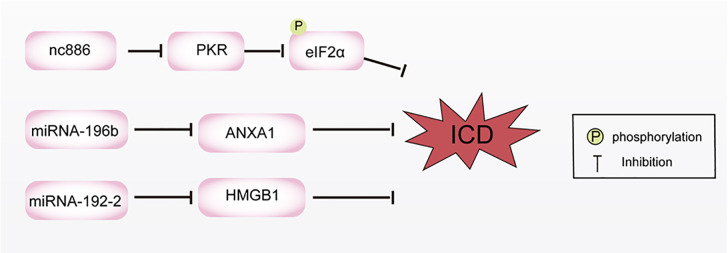
Non coding RNA regulates the mechanism of immunogenic cell death (ICD) by inhibiting calreticulin (CALR) exposure and the expression of annexin A1 (ANXA1) and high mobility group box-1 (HMGB1). The high expression of *nc886* inhibits CALR exposure, hinders PKR catalysis of eIF2α phosphorylation, and subsequently inhibits ICD in cholangiocarcinoma. *miRNA-196b* directly inhibits ANXA1 expression by binding to the non-translated region of the ANXA1 mRNA transcript. *miRNA-129-2* can also regulate the expression of HMGB1 through methylation.

In summary, there is a close interaction between ncRNAs and ICD. Through in-depth research on the mechanism of action of ncRNAs in the ICD process and their potential application value in anti-tumor immune responses, important theoretical basis and technical support can be provided for the development of new immunotherapy methods (**Figure 5**).

### 4.4 Crosstalk of intracellular signaling pathways

The molecular mechanism of inducing ICD is a complex and delicate process, involving the crosstalk of multiple signaling pathways in cells and the interaction between molecules. These pathways jointly regulate the development of ICD, so that the body can effectively deal with risk factors. Current studies have found that ICD interacts with NF-κB, STING, mitogen-activated protein kinase (MAPK), and other signaling pathways, which play an important role in the treatment of MM, breast cancer, pancreatic ductal adenocarcinoma (PDAC), and other tumors.

**Figure 6:**
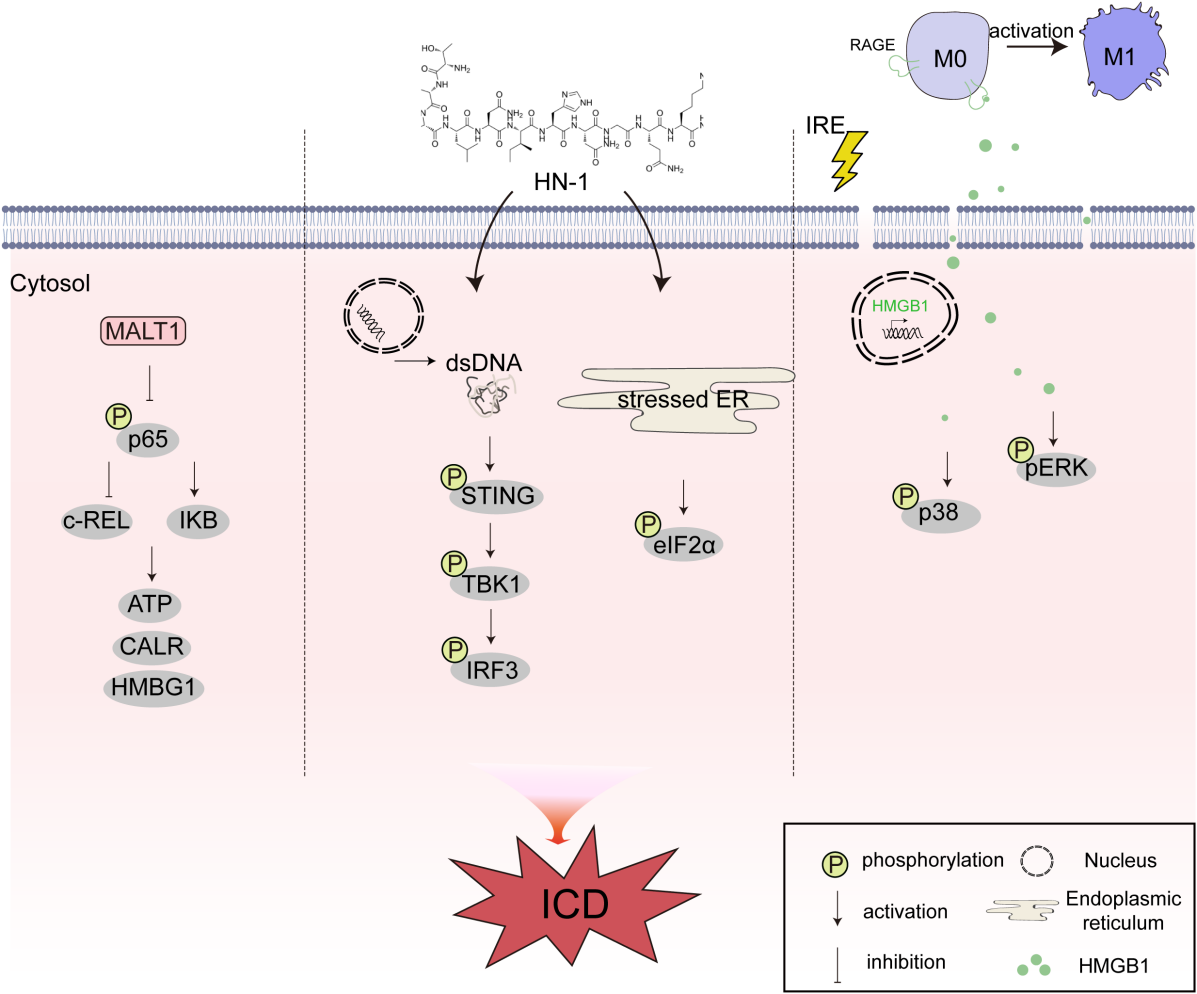
The crosstalk between immunogenic cell death (ICD) and different intracellular signaling pathways of nuclear factor kappa-B (NF-κB), stimulator of interferon genes (STING), and mitogen-activated protein kinase (MAPK). MALT1 can suppress the NF-κB signaling pathway and induce ICD in myeloma. HN-1 treatment induces the release of double-stranded DNA (dsDNA) and triggers ICD by activating the STING pathway. IRE induces ICD in tumor cells by increasing the synthesis and secretion of damage-associated molecular patterns (DAMPs). Additionally, IRE can increase the release of HMGB1 by binding to RAGE. HMGB1 activates the MAPK p38 pathway and upregulates the phosphorylation levels of MAPK-p38 and MAPK-ERK.

Yao et al. found that inhibiting mucosa-associated lymphoid tissue lymphoma translocation protein 1 (MALT1) could suppress the NF-κB signaling pathway and induce ICD in myeloma. After MALT1 depletion, the activation of NF-κB was hindered, resulting in decreased phosphorylation of p65 and reduced c-REL in the nucleus, as well as increased expression of IκB. Additionally, the inhibition of MALT1 increased the release of CALR and ATP, and upregulated the expression of HMGB1, thereby triggering ICD-related immune activation and enhancing the cytotoxicity of CD8^+^ T cells *in vitro*. These findings not only contribute to understanding the pathogenesis of multiple myeloma but also provide a compelling basis for the development of novel clinical strategies for multiple myeloma (130). Our previous study found that Hainanenin-1 (HN-1) could induce ICD in triple-negative breast cancer cell lines and trigger DC maturation, increasing the infiltration of anti-tumor immune cells CD4^+^ and CD8^+^ T lymphocytes. Moreover, the study found that HN-1 treatment induced the release of double-stranded DNA and triggered ICD by activating the STING pathway. The activation of the STING signaling pathway involves several key steps, including cyclic dinucleotide sensing and TBK1/IRF3 activation. When abnormal DNA in the cytoplasm is recognized, cyclic guanosine monophosphate-adenosine monophosphate synthase (cGAS) is activated and catalyzes the synthesis of cyclic guanosine monophosphate-adenosine monophosphate (cGAMP). As a second messenger, cGAMP can bind and activate the STING protein, which is the first step in the activation of the STING signaling pathway. After STING is activated, it translocates to the Golgi apparatus, recruits TBK1 kinase and induces the phosphorylation of IRF3. Compared with the control group, the phosphorylation level of STING was higher in the HN-1 treatment group, and the phosphorylation of STING-related downstream indicators, such as IRF3 and TBK1, was significantly upregulated. HN-1 treatment also increased the phosphorylation of elF2α, a marker of ER stress and ICD. Knockdown of STING by si-STING inhibited the ICD response induced by HN-1. These results suggest that HN-1-induced ICD activates the STING pathway in triple-negative breast cancer cells (131). Irreversible electroporation is an effective method for treating PDAC. He et al. found that irreversible electroporation induces ICD in tumor cells by increasing the synthesis and secretion of DAMPs. Additionally, irreversible electroporation can increase the release of HMGB1 by binding to the receptor of advanced glycation end-product. HMGB1 activates the MAPK p38 pathway and upregulates the phosphorylation levels of MAPK-p38 and MAPK-ERK, leading to an increase in the expression of M1 markers in macrophages. HMGB1 release inhibitors, advanced glycation end-product receptor inhibitors, and MAPK p38 signaling pathway inhibitors can inhibit M1 polarization. In summary, this study reveals that irreversible electroporation can induce ICD in tumor cells by releasing DAMPs. The HMGB1 released by tumor cells after irreversible electroporation treatment promotes M1 macrophage polarization by activating MAPK-p38, and the activation of the MAPK-ERK signaling pathway can positively feedback and enhance M1 macrophage polarization. This study provides a fundamental principle for the combination of irreversible electroporation and immunotherapy in the treatment of PDAC (132). In conclusion, the regulatory relationship between signaling pathways and ICD is an important research topic in the field of biology. Understanding the detailed molecular mechanism of ICD is crucial for the development of ICD-based immunotherapy. Future research should further explore the molecular mechanism of ICD and develop more effective ICD inducers to promote the development of anti-tumor immunotherapy (**Figure 6**).

## 5 Tumor microenvironment and ICD

The tumor microenvironment is a complex ecosystem, including a variety of immune cells, stromal cells, and soluble factors. These components interact with each other and jointly affect the occurrence of ICD and activation of the immune response. ICD also activates the innate and adaptive immune systems to kill tumors by reprogramming the tumor microenvironment (133). Therefore, when developing immunotherapy based on ICD, it is necessary to fully consider the impact of the tumor microenvironment and take corresponding measures to optimize its immunogenicity.

In the tumor microenvironment, macrophages are key immunomodulatory cells, which can be polarized into M1 (anti-tumor) or M2 (pro-tumor) according to the signals of the microenvironment (134). The release of DAMPs and cytokines during ICD can affect the polarization of macrophages, promote the generation of M1 macrophages, and then enhance the anti-tumor immune response (135). For example, HMGB1 combined with TLR4 can activate macrophages to produce pro-inflammatory cytokines such as IL-12 and further promote the Th1 immune response (136). Regulatory T cells (Tregs) play a role in inhibiting the immune response in the tumor microenvironment, thus helping tumor cells escape immune surveillance. ICD can inhibit the function of Tregs by activating effector T cells and increasing the production of IFN-γ and other cytokines, thereby breaking the immunosuppressive state and enhancing anti-tumor immunity (137, 138). In addition, some ICD inducers directly act on Tregs and promote their apoptosis. For example, OXP treatment of Hep-2 cells promoted the production of IL-6 and TNF-α in the supernatant, while inhibiting the activation and stability of Tregs, and induced the production of CALR, ATP, and HMGB1, which inhibited the growth of Hep-2 cells and promoted ICD (139). In addition, DCs play a key role in ICD. When ICD occurs in tumor cells, ectopic CALR (as an “eat-me” signal) promotes DCs or their precursor cells to phagocytize dead or dying tumor cells, thus providing a rich source of antigens, which may then be presented to MHC-I molecules (140). Dying cells release danger signals and chemokines, and the release of related DAMPs triggers the activation and maturation of DCs. APCs are activated while ingesting dead cells, which promotes cross-talk with CD8^+^ T cells and provides a finely regulated signal for subsequent ICD (141). In addition, the anthracycline drug MTX can enhance the phagocytosis of DCs (142). Anthracycline drugs inhibit the protein phosphatase 1/GADD34 complex by inducing ER stress, and then cause rapid phosphorylation of eIF2α, which enables CALR to translocate to the cell surface and activate DCs at the same time. This promotes the phagocytosis by DCs of tumor cells after anthracycline treatment, which causes ICD and enhances the anti-tumor effect *in vivo* (23).

ICD can activate T cells by stimulating the maturation of DCs, and further activate adaptive anti-tumor immunity. The fate of T cells induced by ICD warrants further in-depth study. When T cells undergo an immune response, they form memory T cells, which rapidly react to the same antigen in the future, thus realizing long-term immune monitoring. Whether ICD can induce T cells to transform into memory T cells and realize long-term immunity may be systematically studied in the future using advanced technologies such as single cell sequencing and spatial transcriptomics. Single-cell RNA sequencing technology can be used to systematically analyze T cells in different states, including activated T cells, effector T cells, and memory T cells, and transcriptomics can be used to study the gene expression of T cells in different states. By sequencing and analyzing the gene expression profiles of these cells, we may attain greater insight into the gene expression patterns and differences between T cells in different states, thus revealing their molecular mechanisms. A systematic study on the transformation of T cells into memory T cells induced by ICD through single-cell RNA sequencing and transcriptome technology will help to further understand the immune response mechanism and the formation of immune memory, which will provide a theoretical basis for the development of new immunotherapy strategies.

## 6 Summary and outlook

ICD was first described in 2005 (143) and since then, cancer immunotherapy has progressed significantly. It is now well-established that chemotherapy drugs, viruses, epigenetic modifications, and physical stimuli can all induce ICD in tumor cells. The powerful anti-tumor immunity initiated by ICD inducers, as well as the combination of traditional chemotherapy, RT, and other ICD induction methods with immunotherapy, provide unlimited possibilities for the clinical application of ICD inducers. As research progresses further, it is likely that even more factors that can induce ICD in tumor cells will be discovered. Making full use of the available treatment methods will provide more effective treatment options for cancer.

Although ICD has shown great potential in anti-tumor immunotherapy, its clinical application still faces many challenges. First, only a small number of drugs can induce ICD in clinical practice, and safe and effective alternative chemotherapy drugs to induce ICD are needed. The existing methods to identify such drugs are relatively limited and species-specific. There is an urgent need to develop high-throughput screening methods to screen a large number of ICD inducers (63, 144, 145). Future research should further explore the molecular mechanisms regulating ICD to provide a theoretical basis for the development of new ICD inducers. Second, with the active expansion of the ICD field, there are differences among tumor types and individual patients, leading to inconsistent ICD induction effects. Some patients may be unable to produce effective immune response to ICD inducers due to the weak function of the autoimmune system or the special biological characteristics of tumors. Combining biomarkers (from next generation sequencing, multiplex immunofluorescence, and flow cytometry) with artificial intelligence to quantitatively assess the relative degree of ICD and thereby optimize drug selection is an exciting future research direction. In terms of security, ICD induction may be accompanied by severe side effects, such as systemic inflammatory responses and autoimmune diseases. These side effects not only impair patients’ quality of life but may also lead to treatment intolerance, thereby compromising treatment continuity and therapeutic outcomes. Additionally, conventional drug administration strategies may diminish the efficacy of combination therapy involving ICD inducers and immunotherapy. Such strategies could cause damage to normal cells and induce cytotoxicity. In clinical application, it is necessary to maintain the balance between the efficiency and toxicity of ICD inducers. It is necessary to ensure that the inducers can effectively induce tumor cells to ICD, and to minimize its toxic effect on normal tissues and cells, which puts forward high requirements for the design of drug administration strategies. This is also the safety problem to be solved in the clinical application of ICD inducers, and the dosage of drugs therefore needs to be carefully considered to avoid toxicity and drug resistance. The careful selection of appropriate drugs, *in vivo* assessment methods, and dosages will all determine the success of ICD in clinical treatment plans and the therapeutic effects on tumors.

ICD inducers are of great significance in tumor treatment by activating the body’s anti-tumor immunity and generating long-term anti-tumor effects. Future research directions in ICD inducers and their mechanisms may include: continuing to screen and discover new ICD inducers, which can be done by screening from natural products, synthetic compound libraries, etc., to find drugs with higher activity, lower toxicity, and better targeting. For example, exploring more components from plants and microorganisms and tapping into their potential to induce ICD. In addition, consider structural modification and optimization of existing ICD inducers to improve their efficacy and safety. For instance, chemically modify classic ICD inducers such as anthracyclines and oxaliplatin to enhance their ability to induce ICD and reduce side effects. Develop more effective drug delivery systems to increase the concentration and targeting of ICD inducers in tumor tissues and reduce damage to normal tissues. This can be achieved by using nanocarriers, liposomes, and other methods to precisely deliver ICD inducers to tumor sites. Strengthening preclinical and clinical research on the combined application of ICD inducers and immunotherapy, and exploring the combined application effects of ICD inducers with immune checkpoint inhibitors, cytokines, adoptive cell therapy, and other immunotherapies, will help to develop more effective anti-tumor treatment strategies. For example, the combination of chemotherapy drugs and PD-1 inhibitors has become one of the standard treatment regimens for many tumors. Chemotherapy drugs induce ICD, and then PD-1 inhibitors are used to relieve immune suppression and promote T cells to kill tumor cells. In addition, low-dose chemotherapy or RT-induced ICD can increase the sensitivity of PD-1, making T cells more effective and targeted to tumor cells. This not only improves the treatment effect but also reduces the immune evasion of tumor cells, thereby achieving more effective tumor treatment. With the advancement of technology and in-depth research, we can expect more data to provide theoretical support and practical guidance for this treatment method. Tumor vaccines based on ICD are also a promising personalized treatment strategy. By collecting tumor cell fragments during the ICD process or using genetic engineering techniques to express specific tumor antigens, personalized vaccines targeting the patient’s specific tumor antigens can be prepared to activate their immune system and generate specific anti-tumor immune responses, thereby achieving specific tumor treatment. Future research could employ high-throughput screening to preliminarily identify potential ICD inducers from large compound libraries, providing candidate molecules for further mechanistic studies. Proteomic and transcriptomic profiling would comprehensively delineate intracellular protein dynamics and gene expression alterations during ICD induction, thereby identifying novel therapeutic targets and regulatory networks. Gene editing technologies can elucidate critical signaling cascades in ICD, offering a theoretical foundation for developing precision combination therapies.

By addressing the issues discussed and gaining a deeper understanding of the inducing factors and molecular mechanisms of ICD, we can expect to apply ICD to more types of tumor treatment, providing more effective treatment options, and better quality of life for cancer patients. We have every reason to believe that ICD-based immunotherapy will become one of the important means of anti-tumor treatment in the future. It will not only provide new methods for the clinical treatment of cancer, but may also offer new applications in various life science fields such as virology, immunology, and clinical medicine.

## Glossary

ICD: Immunogenic cell death
CALR: calreticulin
HSPs: heat-shock proteins
ATP: adenosine triphosphate
HMGB1: high mobility group box-1
NPS: Nano-pulse stimulation
RT: radiotherapy
PSs: photosensitizers
PDT: photodynamic therapy
PTT: photothermal therapy
PDIA3: protein disulfide isomerase family A member 3
IFN-I: type I interferon
CXCL9: C-X-C motif chemokine ligand 9
IL-1β: Interleukin-1β
IL-6: Interleukin-6
ANXA1: annexin A1
DAMPs: damage-associated molecular patterns
DCs: dendritic cells
CTLs: cytotoxic T lymphocytes
IFNs: interferons
DOX: doxorubicin
MTX: mitoxantrone
EPI: epirubicin
ER stress: endoplasmic reticulum stress
eIF2α: eukaryotic translation initiation factor 2α
NK: natural killer
IDO1: indoleamine 2,3-dioxygenase-1
PERK: protein kinase RNA-like endoplasmic reticulum kinase
MAPK: mitogen-activated protein kinase
STING: stimulator of interferon genes
DAS: dasatinib
OXP: oxaliplatin
DCs: dendritic cells
EGFR: epidermal growth factor receptor
FOLFIRI: folinic acid, bolus/continuous fluorouracil, and irinotecan
NDV: Newcastle disease virus
MV: measles virus
HSV-1: herpes simplex virus type 1
H-1PV: parvovirus H1
SCC: squamous cell carcinoma
PDAC: pancreatic ductal adenocarcinoma
HDACs: histone deacetylase
APCs: antigen-presenting cells
TNF: tumor necrosis factor
TUSC2: tumor suppressor candidate 2
HPD: hematoporphyrin derivative
LLC: Lewis lung cancer
HPPH: 2-[1-hexyloxyethyl]-2-devinyl pyropheophorbide-a
PEG-b-cPPT: polyethylene glycol-b-cationic polypeptide
IND: indoximod
NF-κB: nuclear factor kappa-B
BZM: bortezomib
IRF3: interferon regulatory factor 3
MHC-I: major histocompatibility complex I
VTCN1: V-set domain-containing T cell activation inhibitor 1
ERO1A: endoplasmic reticulum oxidoreductase 1 alpha
STC1: stanniocalcin 1
ROS: reactive oxygen species
PERK: Protein kinase RNA-like endoplasmic reticulum kinase
IRE1α: inositol-requiring enzyme 1-α
XBP1: X-box protein 1
TCy5: thio-pentamethine cyanine dye
AsC: Ascomylactam C
MTE: marsdenia tenacissima extract
cPLA2: calcium-dependent phospholipase A2
US: ultrasound
PE: pinellia pedatisecta schott extract
ncRNAs: non-coding RNAs
PDAC: pancreatic ductal adenocarcinoma
MALT1: Mucosa-associated lymphoid tissue lymphoma translocation protein 1
HN-1: Hainanenin-1
Tregs: Regulatory T cells
ADCs: antibody-drug conjugates
T-DM1: trastuzumab emtansine
SAC: spindle assembly checkpoint
TACC3: transforming acidic coiled-coil containing 3
PBD: pyrrolobenzodiazepine
GPC2: glypican 2
cGAS: monophosphate-adenosine monophosphate synthase
cGAMP: catalyzes the synthesis of cyclic guanosine monophosphate-adenosine monophosphate.
